# *Staphylococcus epidermidis* Biofilms Have a High Tolerance to Antibiotics in Periprosthetic Joint Infection

**DOI:** 10.3390/life10110253

**Published:** 2020-10-24

**Authors:** John A. Koch, Taylor M. Pust, Alex J. Cappellini, Jonathan B. Mandell, Dongzhu Ma, Neel B. Shah, Kimberly M. Brothers, Kenneth L. Urish

**Affiliations:** 1 Arthritis and Arthroplasty Design Group, Department of Orthopaedic Surgery, University of Pittsburgh, Pittsburgh, PA 15260, USA; j.koch@pitt.edu (J.A.K.); tmp5561@psu.edu (T.M.P.); alc272@pitt.edu (A.J.C.); jbm42@pitt.edu (J.B.M.); dom13@pitt.edu (D.M); kmb227@pitt.edu (K.M.B.); 2 Department of Infectious Diseases and Microbiology, University of Pittsburgh, Pittsburgh, PA 15260, USA; 3 Division of Infectious Disease, Department of Internal Medicine, University of Pittsburgh Medical Center, Pittsburgh, PA 15260, USA; shahnb@upmc.edu; 4 The Bone and Joint Center, Magee Womens Hospital of the University of Pittsburgh Medical Center, Pittsburgh, PA 15213, USA; 5 Department of Bioengineering, University of Pittsburgh, Pittsburgh, PA 15260, USA; 6 Clinical and Translational Science Institute, University of Pittsburgh, Pittsburgh, PA 15260, USA

**Keywords:** periprosthetic joint infection, infection, antibiotic resistance, PJI, *s. epidermidis*, arthroplasty

## Abstract

Both *Staphylococcus aureus* and *Staphylococcus epidermidis* are commonly associated with periprosthetic joint infections (PJIs). The treatment of PJI can be challenging because biofilms are assumed to have an increased intolerance to antibiotics. This makes the treatment of PJI challenging from a clinical perspective. Although *S. aureus* has been previously demonstrated to have increased biofilm antibiotic tolerance, this has not been well established with *Staphylococcus epidermidis*. A prospective registry of PJI *S. epidermidis* isolates was developed. The efficacy of clinically relevant antibiotics was quantified against these isolates. *S. epidermidis* planktonic minimum inhibitory concentration (MIC) and minimum bactericidal concentration (MBC) were collected using clinical laboratory standard index (CLSI) assays for eight antibiotics (doxycycline, vancomycin, daptomycin, clindamycin, rifampin, nafcillin, and trimethoprim/sulfamethoxazole). Mature biofilms were grown in vitro, after which minimum biofilm inhibitory concentration (MBIC) and minimum biofilm bactericidal concentration (MBBC) were quantified. Only rifampin and doxycycline had a measurable MBIC across all tested isolates. Based on MBBC, 64% of *S. epidermidis* biofilms could be eliminated by rifampin, whereas only 18% by doxycycline. *S. epidermidis* biofilm was observed to have a high tolerance to antibiotics as compared to planktonic culture. Isolate biofilm antibiotic tolerance varied to a larger degree than was seen in planktonic cultures.

## 1. Introduction

*Staphylococcus epidermidis* and *Staphylococcus aureus* are the two most commonly found microorganisms associated with periprosthetic joint infection (PJI) [1,2]. It has been established that *S. aureus* will rapidly form biofilms in implant infections [3,4,5]. *S. epidermidis* has also been demonstrated to rapidly form a biofilm on surgical material in less than 12 h of growth [6]. These infections are challenging to treat due to the fact that bacterial cells in biofilms have a higher tolerance to antibiotic therapy [2]. Increased complications in other biofilm related infections, such as prosthetic valve endocarditis, have been observed with coagulase-negative staphylococci species like *S. epidermidis* [7]. Similar biofilm tolerance has also been demonstrated in a variety of bacteria [8,9,10], and many drug tolerance mechanisms have been proposed.

Standard antibiotic tolerance assays focus on measuring susceptibility against planktonic strains; however, *S. epidermidis* and many other bacteria primarily exist in the biofilm state during infection [11]. Although this method accurately quantifies planktonic antibiotic tolerance, it fails to measure antibiotic activity against biofilms. It has been previously demonstrated that biofilms have significantly decreased antibiotic susceptibility [2,12,13,14]. There are a variety of mechanisms for this increased antibiotic tolerance, which includes bacterial persisters [15], decreased metabolism [16], and the shielding effect of extracellular polymeric substances [17]. From a clinical perspective, standard antibiotic susceptibility testing provides little use for the treatment of established biofilms in cases of PJI. By in vitro testing of isolates derived from PJI and cultured as biofilms, we can more accurately determine biofilm antibiotic tolerance.

There are a limited number of studies that have evaluated the activity of antibiotics against *S. epidermidis* biofilms specifically using PJI clinical isolates [18,19]. Previous studies have demonstrated *S. epidermidis* biofilm has an increased tolerance to antibiotics and required combination antibiotic therapy to achieve an antimicrobial effect on laboratory strains and clinical isolates from a catheter-related infection [19]. To address this paucity of data, we developed a prospective clinical isolate registry of total knee arthroplasty (TKA) PJI samples to quantify the sensitivity of different antibiotics to clinical isolates of *S. epidermidis* in vitro in cultured biofilms. Both planktonic and biofilm MIC and MBC of a panel of commonly administered antibiotics were quantified across all isolates. By comparing biofilm and planktonic tolerance, the objective was to determine the differences in biofilm antibiotic sensitivity.

## 2. Results

### 2.1. S. epidermidis Antibiotic Sensitivity Is Lower in Planktonic Cultures in Comparison to Mature Biofilms

**Figure 1:**
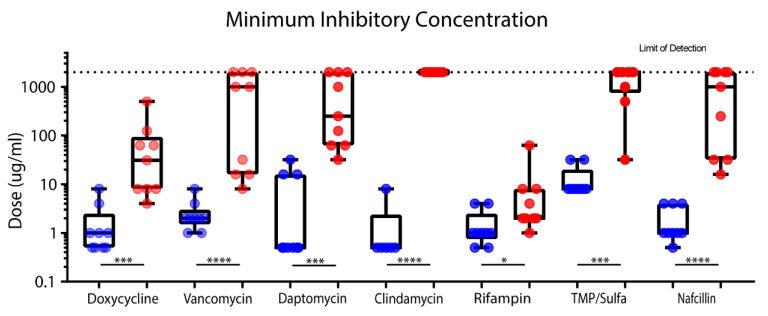
Mature biofilms MBIC (red) demonstrate decreased inhibition sensitivity to clinically significant antibiotics when compared to planktonic MIC (blue). PrestoBlue staining assay was performed to compare MIC and MBIC. Statistical comparison of planktonic MIC and biofilm MIC (MBIC) was conducted using a Mann–Whitney test. Significance is indicated (**** *p* < 0.0001, *** *p* = 0.0001, ** *p* < 0.001, * *p* < 0.05), and most antibiotics demonstrated significant increases in MIC.

Using a clinical laboratory standard index (CSLI) assay protocol and an in vitro biofilm assay, we quantified variations in planktonic MIC and planktonic MBC of clinically relevant antibiotics across 11 different strains of *S. epidermidis*. Clindamycin and daptomycin all showed larger variations (>2 log difference) in planktonic MIC across all isolates, while clindamycin, trimethoprim/sulfamethoxazole (TMP/sulfa), vancomycin, and rifampin displayed smaller variation (~1 log spread) (Figure 1). In contrast to their planktonic counterparts, mature biofilms demonstrated an increased tolerance to clinically significant antibiotics, with this difference statistically significant in all eight antibiotics tested (Figure 1).

### 2.2. Variations in Minimum Bactericidal Concentration Against S. epidermidis Planktonic Cultures and Mature Biofilms

**Figure 2:**
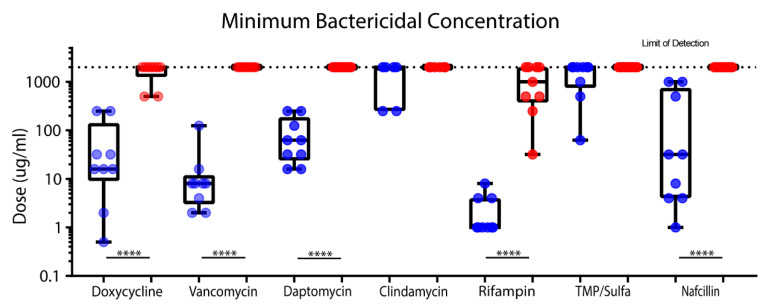
Comparison of bactericidal activity of clinically relevant antibiotics against planktonic MBC (blue) and biofilm MBBC (red) of the tested antibiotics against *S. epidermidis*. Serial dilution and CFU counts were used to compare MBC and MBBC. A Mann–Whitney significance test was used (**** *p* < 0.0001). Nearly all antibiotics demonstrated significant results, with a *p*-value of less than 0.0001; however, clindamycin and TMP/sulfa were equally ineffective at eradication of either planktonic or biofilm cultures.

Following the same CLSI protocol as described above, we quantified MBIC and biofilm MBBC using the same clinically relevant antibiotics and strains as described in Figure 1. Daptomycin and rifampin had a relatively low variation in planktonic MBC (1 log spread) compared to all other antibiotics (> 2 log spread) (Figure 2). Rifampin showed the greatest efficacy against planktonic MBC, with doses ranging from 0.5 to 8 µg/mL. Virtually all antibiotics demonstrated significant differences between planktonic and biofilm cultures (six out of eight). Clindamycin and TMP/sulfa were ineffective and unable to eliminate either planktonic or biofilm cultures.

### 2.3. S. epidermidis Biofilms Demonstrate Increased Antibiotic Tolerance

**Figure 3:**
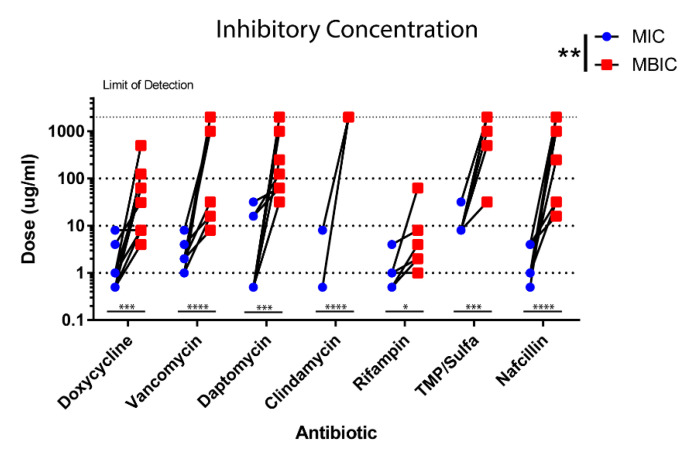
PJI *S. epidermidis* biofilms show increased antibiotic tolerance. Minimum inhibitory concentration was determined for all strains using a PrestoBlue viability assay. Planktonic MIC (blue) and biofilm MIC (red) were compared. MBIC showed a larger variation than seen in the MIC of most strains. Significance is indicated (**** *p* < 0.0001, *** *p* = 0.0001, ** *p* < 0.001, * *p* < 0.05).

The difference in antibiotic tolerance was tested between biofilm and planktonic states of growth in our clinical strains. In the majority of antibiotics tested, the MBIC of strains showed a larger variation in comparison to the MIC of their planktonic states (Figure 3). This is most likely due to many of the antibiotics like clindamycin failing to achieve MBIC levels in biofilm cultures, with many biofilms right at the limit of detection for our assays. However, both rifampin and doxycycline achieved MBIC in all tested strains, and a larger variation in MBIC was observed in biofilms in comparison to MIC in planktonic strains (2 log spread) (Figure 3).

### 2.4. Multiple Antibiotics Fail to Eliminate S. epidermidis Biofilms

**Figure 4:**
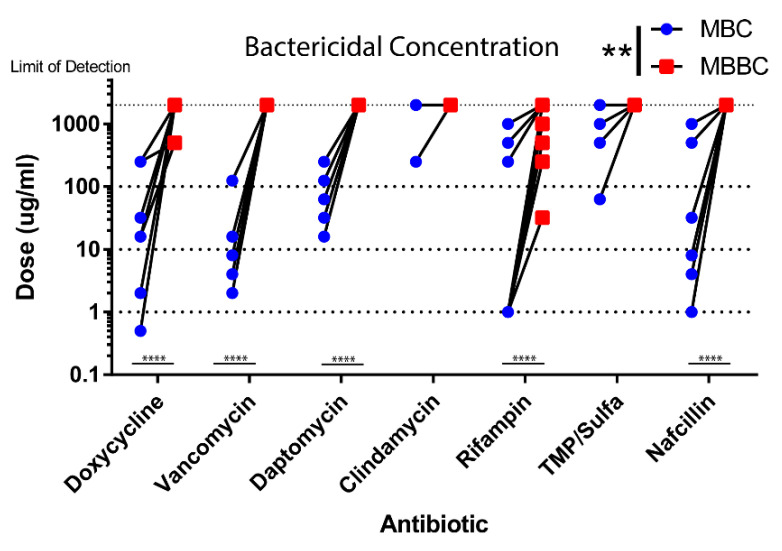
PJI *S. epidermidis* biofilms show increased antibiotic tolerance. Minimum inhibitory concentration was determined for all strains using a PrestoBlue viability assay. Planktonic MIC (blue) and biofilm MIC (red) were compared. MBIC showed a larger variation than seen in the MIC of most strains. Significance is indicated (**** *p* < 0.0001, *** *p* = 0.0001, ** *p* < 0.001, * *p* < 0.05).

We assessed the ability of our clinically relevant antibiotics to eradicate *S. epidermidis* biofilms. Minimum bactericidal concentrations were determined from planktonic and biofilm strains by CFU analysis. Many antibiotics failed to achieve MBBC levels similar to the observations in our MBIC studies. Biofilms were extremely tolerant to all antibiotics in comparison to their planktonic states (Figure 4). All biofilm strains were completely resistant to clindamycin as observed in Figure 3 for our MIC studies and also failed to achieve MBBC despite effectiveness when tested in their planktonic state. Only rifampin and doxycycline were able to achieve MBBC, with values ranging from 32 to 2000 µg/mL (Figure 4). In comparison to rifampin, clinical strains in the biofilm state were much more resistant to doxycycline and had much higher MBBC values.

## 3. Discussion

Guidelines for the treatment of PJI provide treatment dosages that are based on antibiotic susceptibility of bacteria in its planktonic state alone. These dosages, however, may not be representative of the biofilm state these infections exist in, and this in turn could lead to suboptimal clinical outcomes [11,20]. In PJI and other implant related infections, *S. epidermidis* is highly pervasive and difficult to eradicate, primarily due to its propensity to develop a mature biofilm [20,21]. Due to this discrepancy between guideline recommendations and clinical application, our goal was to identify variations in antibiotic susceptibility between typical planktonic cultures and mature biofilms of *S. epidermidis* isolates in the setting of clinical infection. We observed significantly reduced susceptibility across all *S. epidermidis* isolates for most of our tested antibiotics (Table S1), with only rifampin and doxycycline demonstrating any bactericidal effect against established biofilms.

Biofilm susceptibility was reduced across all tested antibiotics, reinforcing the concept of nonspecific tolerance mechanisms suggested by our previous work [14]. Rifampin and daptomycin retained the most inhibitory effectiveness for treatment of biofilms, and doxycycline had a greater reduction in inhibition, while all other antibiotics varied substantially. Antibiotic tolerance of biofilm-released cells have been demonstrated, and this intermediate phenotype has been shown to statistically reduce antibiotic sensitivity in comparison to planktonic cells [22]. Recently, vancomycin, rifampin, and gentamicin have been shown to be effective against *S. epidermidis* biofilms [22]. Rifampin monotherapy is highly discouraged, however, due to the development of antibiotic resistance observed in staphylococcal infections. Combination therapy using fusidic acid has been proven to have moderately increased effectiveness for treatment of staphylococcal infections [23]. Rifampin is traditionally combined with cefazolin or vancomycin for treatment of PJI [21]. Daptomycin, a recently developed lipopeptide, has demonstrated antimicrobial activity independent of bacterial metabolism [14,24,25]. By disrupting the bacterial cellular membrane resulting in cellular lysis, daptomycin has been shown to be effective against *S. aureus* and *S. epidermidis* biofilms [14,24,25]. However, in the *S. epidermidis* strains tested for this study, doxycycline demonstrated a similar efficacy to daptomycin in biofilm inhibition, as well as a slightly improved bactericidal efficacy. This data is derived entirely from in vitro studies, but our goal was not to recommend certain antibiotics in the clinical treatment of PJI. Instead, our goal was to demonstrate the differences in antibiotic tolerance between planktonic and biofilm clinical isolates.

Biofilm development and maturation introduces complexities typically not seen in planktonic culture, which complicates comparisons for antibiotic susceptibility. We included serial dilutions of untreated biofilms to obtain a more accurate way to quantify biofilm density. Strain diversity could also account for variation within planktonic and biofilm MIC and MBC. Biophysical characteristics, such as biofilm extracellular secretions or metabolic output, were not measured. Most *S. epidermidis* biofilm studies measure antibiotic susceptibility within 24–48 h of antibiotic exposure. *S. aureus* has been demonstrated to have increased susceptibility to antibiotics with longer exposure periods of up to five days [26]. For these studies, we chose to treat each growth condition for only 24 h to clearly differentiate between planktonic and biofilm antibiotic susceptibility. By narrowing the scope of our investigation, we demonstrated a clear decrease in antibiotic sensitivity against *S. epidermidis* biofilm. All antibiotics failed to eradicate *S. epidermidis* biofilm within the recommended dosing ranges. Rifampin, the optimal anti-biofilm antibiotic tested, could only effectively eradicate the biofilm in vitro at doses associated with increased toxicity.

*S. epidermidis*, as well as many other pathogens, rapidly forms biofilms in PJI. These biofilms have been demonstrated to have extremely high antibiotic tolerance. Our data reinforces this notion and suggests that antibiotic treatment of *S. epidermidis* in orthopedic infections alone is insufficient to eradicate these biofilms. Surgical debridement with antimicrobial irrigation and the host immune response are critical for clearance of orthopedic related biofilm infections.

Our goal is not to undermine the use of standard planktonic antibiotic susceptibility testing. These standards provide valuable clinical data regarding antibiotic resistance; however, it fails to consider the antibiotic resistance of biofilms common in PJI. This study suggests there is a phenotypic and non-specific change in antibiotic tolerance that occurs between planktonic and biofilm stages in bacterial infections and further demonstrates the capacity of common PJI pathogens to form antibiotic resistant biofilms. In addition, our data suggests that rifampin, doxycycline, and daptomycin demonstrated the highest efficacy against in vitro biofilms. Given that no single antibiotic was bactericidal against biofilm, this adds further evidence to the use of combination therapy with rifampin in implant associated infections [19]. In vivo studies are warranted to confirm clinical application of these results.

## 4. Materials and Methods

### 4.1. Culture Conditions and Bacterial Strains

Frozen isolates were used to inoculate into 5 mL of tryptic soy broth (TSB; Bectin Dickinson and Company, Franklin Lakes, NJ, USA). The conical tube containing the inoculate was incubated overnight at 37 °C with shaking at 250 rpm. After 16 h of incubation, Mueller Hinton broth (MHB; Bectin Dickinson and Company) was used to dilute all strains to 0.5 × 10^6^ CFU/mL using the 0.5 MacFarland Standard (GFS Chemicals, Columbus, OH, USA) in an Infinite M200 Spectrophotometer (Tecan, Männedorf, Switzerland). High throughput methods were used for experiments in sterile, tissue culture treated, flat bottom 96-well plates (Thermo Fisher Scientific, Waltham, MA, USA). All trials were performed in triplicate using fresh, independent inoculates for each culture. Two lab strains of *S. epidermidis* ATTC 12228 [27] and ATTC 35894 [28], as well as nine clinical isolates from PJI in arthroplasty infections were tested. Clinical isolates from PJI patients were obtained from a clinical testing laboratory from cultures prepared on TSB agar slants. *S. epidermidis* clinical isolates were then grown in TSB overnight with shaking and stored in cryotubes at −80 °C in TSB with 10% glycerol to create an isolate library bank. All procedures for this study were followed according to Institutional Review Board (IRB) guidelines and regulations, IRB approval #PRO15070263.

### 4.2. Planktonic Culture MIC and MBC

Sterile tissue culture treated flat bottom 96-well plates were used to treat planktonic *S. epidermidis* at an initial concentration of 0.5 × 10^6^ CFU/mL. A two-fold serial dilution was used to create antibiotic dilutions (doxycycline, vancomycin, daptomycin, clindamycin, rifampin, nafcillin, and trimethoprim/sulfamethoxazole) at a final volume of 100 µL. Penicillin (1000, 500, 250, 125, 62, 31, 16, 8, 4, 2, and 1 µg/mL and untreated control) was used to treat planktonic bacteria. Minimum inhibitory concentration (MIC) was assessed using PrestoBlue viability reagent (Thermo Fisher Scientific) according to the manufacturer’s instructions and read on an Infinite M200 Spectrophotometer (Thermo Fisher Scientific) [29] after 10 min of PrestoBlue exposure. Minimum bactericidal concentration (MBC) was assessed by serial dilution of treated cultures plated on tryptic soy agar (TSA) II CS100 plates with 5% sheep blood. These methods are clinical laboratory standard index (CLSI) assays [11,30]. Agar plates were incubated for 24 h at 37 °C, then a colony forming unit (CFU) analysis was performed. In situations where clinical isolates had a genetic resistance to the class of antibiotics, that specific antibiotic was not tested on that isolate. Planktonic MBC was considered a 99.9% decrease in CFUs of the original bacterial concentration (0.5 × 10^6^ CFU/mL) [31]. 

### 4.3. Mature Biofilms MIC and MBC

*S. epidermidis* biofilms were cultured planktonically in Mueller Hinton broth (MHB) at an original concentration of 0.5 × 10^6^ CFU/mL in sterile tissue culture treated flat bottom wells. After 24 h of static growth, original MHB was discarded and replaced with fresh MHB. After an additional 24 h of growth, MHB was removed and the wells were irrigated with PBS to remove remaining planktonic bacteria. The mature biofilm was then treated with the same antibiotic panel as the planktonic assay. Antibiotic doses were doubled to account for the increased antibiotic tolerance of biofilms (2000, 1000, 500, 250, 125, 62, 31, 16, 8, 4, and 2 µg/mL) and suspended in fresh MHB. After 24 h of antibiotic treatment, the media was removed and the replaced with 100 µL of phosphate buffered saline (PBS). The 96 wells were scraped manually for one minute to dislodge the adhered biofilm and homogenized within the PBS. The scraping process used autoclaved 0.1–10 µL micropipette tips held in their wafer rack with tape. Pipette tips were simultaneously inserted into all wells to thoroughly dislodge the biofilm. Minimum biofilm bactericidal concentration (MBBC) was determined using serial dilution on agar plates. The MBBC was a 99.9% reduction from the CFU assay of the serial dilution of the control wells to adjust for continued growth of the biofilm over the 48 h. Minimum biofilm inhibitory concentration (MBIC) was determined using PrestoBlue viability assay. Biofilms were exposed to PrestoBlue for one hour before being quantified using an Infinite M200 spectrophotometer to allow for a definitive color change.

### 4.4. Statistical Analysis

Antibiotic tolerance was compared between planktonic and established biofilms from nine isolates chosen from our PJI clinical isolate library and two laboratory strains. All graphical and statistical analysis was performed using Prism 7.0 (GraphPad, La Jolla, CA, USA) using the same statistical tests as previously described for *S. aureus* [14]. Data was analyzed using a D’Agostino & Pearson normality test. A Mann–Whitney test was used to compare two groups with non-parametric data. Method sample analysis was performed using a Wilcoxon rank test, and multiple group variance testing was performed using a Kruskal–Wallis test with a Dunn’s multiple comparisons post-test.

## Supplementary Materials

The following are available online at https://www.mdpi.com/2075-1729/10/11/253/s1, Table S1. Antibiotic sensitivity of the isolates for each of the antibiotics tested. S = sensitive; R = resistant.

## Abbreviations

PJI	Periprosthetic joint infection
M(B)IC	Minimum (biofilm) inhibitory concentration
MB(B)C	Minimum (biofilm) bactericidal concentration
