# Supplementary material for: Staphylococcus epidermidis Biofilms Have a High Tolerance to Antibiotics in Periprosthetic Joint Infection
Source: Life (Basel). 2020 Oct 24;10(11):253. doi: 10.3390/life10110253 (PMC7693748; doi:10.3390/life10110253)
Supplement: Supplementary file 1 [file life-10-00253-s001.pdf]

life

Supplementary Material of *Staphylococcus epidermidis* Biofilms Have a High Tolerance to Antibiotics in Periprosthetic Joint Infection

Table S1. Antibiotic sensitivity of the isolates for each of the antibiotics tested. S = sensitive; R = resistant.

Isolate	Type	Doxycycline	Vancomycin	Daptomycin	Clindamycin	Rifampin	TMP/Sulfa	Nafcillin
ATCC35984	Catheter sepsis	S	S	S	S	S	S	S
ATCC12228		S	S	S	S	S	S	S
Clinical 1	Sonicate	S	S	S	S	S	S	S
Clinical 2	Synovial Fluid	S	S	S	S	S	S	R
Clinical 3	Sonicate	S	S	S	S	S	R	S
Clinical 4	Synovial Fluid	S	S	S	R	R	R	R
Clinical 5	Sonicate	R	S	S	R	S	S	R
Clinical 6	Sonicate	S	S	S	S	S	S	S
Clinical 7	Sonicate	S	S	S	S	S	R	R
Clinical 8	Sonicate	S	S	S	S	S	R	R
Clinical 9	Sonicate	S	S	S	S	S	R	R

Publisher's Note: MDPI stays neutral with regard to jurisdictional claims in published maps and institutional affiliations.

© 2020 by the authors. Licensee MDPI, Basel, Switzerland. This article is an open access article distributed under the terms and conditions of the Creative Commons Attribution (CC BY) license (http://creativecommons.org/licenses/by/4.0/).
